# The chromosomal genome sequence of the tubeworm, *Lamellibrachia columna* Southward, 1991 (Sabellida: Siboglinidae)

**DOI:** 10.12688/wellcomeopenres.25860.1

**Published:** 2026-02-16

**Authors:** Yanan Sun, Jian-Wen Qiu, Chong Chen, José M. Martín-Durán, Graeme Oatley, Elizabeth Sinclair, Eerik Aunin, Noah Gettle, Camilla Santos, Michael Paulini, Haoyu Niu, Victoria McKenna, Rebecca O’Brien, Wellcome Sanger Institute Tree of Life Management, Samples and Laboratory team, Wellcome Sanger Institute Scientific Operations: Sequencing Operations, Wellcome Sanger Institute Tree of Life Core Informatics team, EBI Aquatic Symbiosis Genomics Data Portal Team, Aquatic Symbiosis Genomics Project Leadership

**Affiliations:** 1 Laboratory of Marine Organism Taxonomy and Phylogeny, Institute of Oceanology, Chinese Academy of Sciences, Qingdao, China; 2 Department of Biology, Hong Kong Baptist University, Hong Kong, China; 3 X-STAR, Japan Agency for Marine-Earth Science and Technology (JAMSTEC),, Yokosuka, Kanagawa, Japan; 4 School of Biological and Behavioural Sciences, Queen Mary University of London, London, England, UK; 5 Tree of Life Programme, Wellcome Sanger Institute, Hinxton, England, UK

**Keywords:** Lamellibrachia columna; tubeworm; genome sequence; chromosomal; Sabellida; microbial metagenome assembly

## Abstract

We present a genome assembly from an individual *Lamellibrachia columna* (tubeworm; Annelida; Polychaeta; Sabellida; Siboglinidae). The genome sequence has a total length of 879.73 megabases. Most of the assembly (99.96%) is scaffolded into 15 chromosomal pseudomolecules. The mitochondrial genome has also been assembled, with a length of 16.78 kilobases. Gene annotation of this assembly by Ensembl identified 21 983 protein-coding genes.

## Species taxonomy

Eukaryota; Opisthokonta; Metazoa; Eumetazoa; Bilateria; Protostomia; Spiralia; Lophotrochozoa; Annelida; Polychaeta; Sedentaria; Canalipalpata; Sabellida; Siboglinidae; *Lamellibrachia*; *Lamellibrachia columna* Southward, 1991 (NCBI:txid53616)

## Background

*Lamellibrachia columna* Southward, 1991 belongs to Vestimentifera, a clade of conspicuous tube-building worms in the annelid family Siboglinidae, inhabiting deep-sea hydrothermal vents, cold-water seeps, and organic falls ( Bright & Lallier, 2010). These tubeworms were initially treated as a new order or even phylum due to their highly unusual body plan ( Jones, 1985), but were later shown to be a derived clade within Siboglinidae. The body of a vestimentiferan is divided into four sections: the anterior gill plume, the vestimentum, the trunk, and the opisthosoma. Vestimentiferans lack a digestive system and rely on endosymbiotic bacteria in the trophosome of their trunk for energy and nutrients. The bacteria were obtained from the environment during settlement in each generation, and these symbionts utilise hydrogen sulphide contained in geofluids or the sediment as the energy source for chemosynthesis. *Lamellibrachia* is an early-diverging genus of vestimentiferans with nine recognised species. It can be distinguished from other vestimentiferan genera by the following combination of characteristics ( Southward, 1991): the gill filaments are parallel to the obturaculum and attached around its base; the obturaculum lacks encrustation or ornamentation; there are sheath lamellae outside the branchial lamellae; and the anterior and posterior ends of the vestimental folds do not fuse.

**Figure 1.**
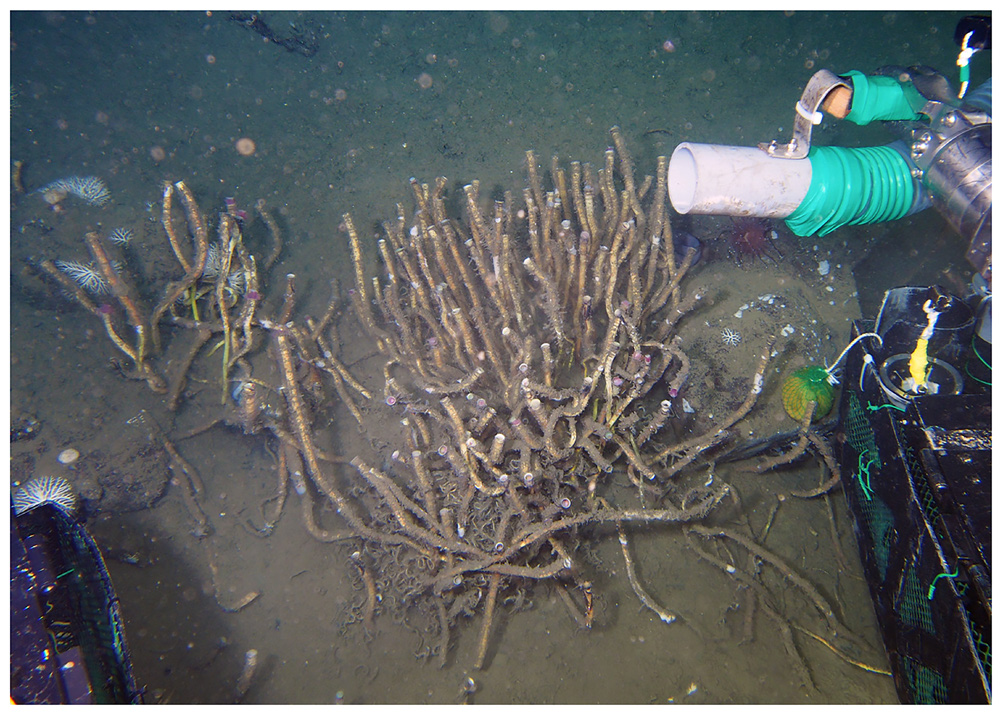
Photograph showing a bouquet of *Lamellibrachia columna* at the Off Hatsushima seep, Samagi Bay.

Southward (1991) described *L. columna* as a new species from the Lau Back-Arc Basin, based on unique features of the shape and size of the tube, the obturaculum, and the number of sheath lamellae, but McCowin *et al.* (2019) commented that only the numbers of sheath and branchial lamellae are distinct features of this species. *L. columna* has been reported from both seep and vent habitats, with a wide distribution from Sagami Bay, Japan, to New Zealand in the Pacific Ocean ( McCowin *et al.*, 2019). *Lamellibrachia* worms from Sagami Bay, Okinawa Trough, and Nankai Trough in Japan were initially split into two clades, L1 and L2, based on the mitochondrial cytochrome *c* oxidase subunit I (COI) gene ( Kojima *et al.*, 2001), with L1 later named as *L. sagami* Kobayashi *et al.* (2015). However, McCowin *et al.* (2019) later showed that both are conspecific with *L. columna*, based on their morphology and the small genetic distances in two mitochondrial genes (16S, COI) between these populations and specimens from the type locality. The *L. columna* individual used for genome sequencing herein was collected from the Hatsushima seep site ( Hashimoto *et al.*, 1989) in Sagami Bay ( Figure 1). The tubes of this species measure up to 2 cm in diameter and typically up to 1 m in length, although in extreme cases they may reach up to 2 m.

The *L. columna* reference genome will contribute to understanding of the molecular basis of the metabolic complementarity between annelid hosts and their symbionts and comparison with other siboglinid holobionts, such as *Riftia pachyptila* ( de Oliveira *et al.*, 2022), *Osedax frankpressi* ( Moggioli *et al.*, 2023) and *Paraescarpia echinospica* ( Sun *et al.*, 2021), will provide information about the diversity of symbioses that allowed vestimentiferans to colonise various deep-sea chemosynthetic habitats.

## Methods

### Sample acquisition

The specimen used for genome sequencing was a *Lamellibrachia columna* (specimen ID QMOUL0000077, ToLID wsLamColu1; Figure 1), collected from Off Hatsushima seep, Sagami Bay, Japan (latitude 35.0156, longitude 139.2239), at a depth of 929 m on 2021-08-22, during HOV (human-occupied vehicle) *Shinkai 6500* dive 1599 on the R/V *Yokosuka* (cruise YK21-15C). The specimen was collected and identified by Chong Chen (Japan Agency for Marine-Earth Science and Technology). The same specimen was used for RNA sequencing.

### Nucleic acid extraction

Protocols for high molecular weight (HMW) DNA extraction developed at the Wellcome Sanger Institute (WSI) Tree of Life Core Laboratory are available on protocols.io ( Howard *et al.*, 2025). The wsLamColu1 sample was weighed and triaged to determine the appropriate extraction protocol. Tissue was homogenised by powermashing using a PowerMasher II tissue disruptor. HMW DNA was extracted using the Manual MagAttract protocol. DNA was sheared into an average fragment size of 12–20 kb following the Megaruptor®3 for LI PacBio protocol. Sheared DNA was purified by automated SPRI (solid-phase reversible immobilisation). The concentration of the sheared and purified DNA was assessed using a Nanodrop spectrophotometer and Qubit Fluorometer using the Qubit dsDNA High Sensitivity Assay kit. Fragment size distribution was evaluated by running the sample on the FemtoPulse system. For this sample, the final post-shearing DNA had a Qubit concentration of 18.1 ng/μL and a yield of 2 353.00 ng.

RNA was extracted from thorax tissue of wsLamColu1 in the Tree of Life Laboratory at the WSI using the RNA Extraction: Automated MagMax™ *mir*Vana protocol. The RNA concentration was assessed using a Nanodrop spectrophotometer and a Qubit Fluorometer using the Qubit RNA Broad-Range Assay kit. Analysis of the integrity of the RNA was done using the Agilent RNA 6000 Pico Kit and Eukaryotic Total RNA assay.

### PacBio HiFi library preparation and sequencing

Library preparation and sequencing were performed at the WSI Scientific Operations core. Libraries were prepared using the SMRTbell Prep Kit 3.0 (Pacific Biosciences, California, USA), following the manufacturer’s instructions. The kit includes reagents for end repair/A-tailing, adapter ligation, post-ligation SMRTbell bead clean-up, and nuclease treatment. Size selection and clean-up were performed using diluted AMPure PB beads (Pacific Biosciences). DNA concentration was quantified using a Qubit Fluorometer v4.0 (ThermoFisher Scientific) and the Qubit 1X dsDNA HS assay kit. Final library fragment size was assessed with the Agilent Femto Pulse Automated Pulsed Field CE Instrument (Agilent Technologies) using the gDNA 55 kb BAC analysis kit.

The sample was sequenced using the Sequel IIe system (Pacific Biosciences, California, USA). The concentration of the library loaded onto the Sequel IIe was in the range 40–135 pM. The SMRT link software, a PacBio web-based end-to-end workflow manager, was used to set-up and monitor the run, and to perform primary and secondary analysis of the data upon completion.

### Hi-C

***Sample preparation and crosslinking***

The Hi-C sample was prepared from 20–50 mg of frozen tissue from the wsLamColu1 sample using the Arima-HiC v2 kit (Arima Genomics). Following the manufacturer’s instructions, tissue was fixed and DNA crosslinked using TC buffer to a final formaldehyde concentration of 2%. The tissue was homogenised using the Diagnocine Power Masher-II. Crosslinked DNA was digested with a restriction enzyme master mix, biotinylated, and ligated. Clean-up was performed with SPRISelect beads before library preparation. DNA concentration was measured with the Qubit Fluorometer (Thermo Fisher Scientific) and Qubit HS Assay Kit. The biotinylation percentage was estimated using the Arima-HiC v2 QC beads.

***Hi-C library preparation and sequencing***

Biotinylated DNA constructs were fragmented using a Covaris E220 sonicator and size selected to 400–600 bp using SPRISelect beads. DNA was enriched with Arima-HiC v2 kit Enrichment beads. End repair, A-tailing, and adapter ligation were carried out with the NEBNext Ultra II DNA Library Prep Kit (New England Biolabs), following a modified protocol where library preparation occurs while DNA remains bound to the Enrichment beads. Library amplification was performed using KAPA HiFi HotStart mix and a custom Unique Dual Index (UDI) barcode set (Integrated DNA Technologies). Depending on sample concentration and biotinylation percentage determined at the crosslinking stage, libraries were amplified with 10–16 PCR cycles. Post-PCR clean-up was performed with SPRISelect beads. Libraries were quantified using the AccuClear Ultra High Sensitivity dsDNA Standards Assay Kit (Biotium) and a FLUOstar Omega plate reader (BMG Labtech).

Prior to sequencing, libraries were normalised to 10 ng/μL. Normalised libraries were quantified again to create equimolar and/or weighted 2.8 nM pools. Pool concentrations were checked using the Agilent 4200 TapeStation (Agilent) with High Sensitivity D500 reagents before sequencing. Sequencing was performed using paired-end 150 bp reads on the Illumina NovaSeq 6000.

### RNA library preparation and sequencing

Libraries were prepared using the NEBNext ^®^ Ultra™ II Directional RNA Library Prep Kit for Illumina (New England Biolabs), following the manufacturer’s instructions. Poly(A) mRNA in the total RNA solution was isolated using oligo(dT) beads, converted to cDNA, and uniquely indexed; 14 PCR cycles were performed. Libraries were size-selected to produce fragments between 100–300 bp. Libraries were quantified, normalised, pooled to a final concentration of 2.8 nM, and diluted to 150 pM for loading. Sequencing was carried out on the Illumina NovaSeq 6000, generating paired-end reads.

### Genome assembly

Prior to assembly of the PacBio HiFi reads, a database of *k*-mer counts ( *k* = 31) was generated from the filtered reads using FastK. GenomeScope2 ( Ranallo-Benavidez *et al.*, 2020) was used to analyse the *k*-mer frequency distributions, providing estimates of genome size, heterozygosity, and repeat content.

The HiFi reads were assembled using Hifiasm ( Cheng *et al.*, 2021) with the --primary option. Haplotypic duplications were identified and removed using purge_dups ( Guan *et al.*, 2020). The Hi-C reads ( Rao *et al.*, 2014) were mapped to the primary contigs using bwa-mem2 ( Vasimuddin *et al.*, 2019), and the contigs were scaffolded in YaHS ( Zhou *et al.*, 2023) with the --break option for handling potential misassemblies. The scaffolded assemblies were evaluated using Gfastats ( Formenti *et al.*, 2022), BUSCO ( Manni *et al.*, 2021) and MERQURY.FK ( Rhie *et al.*, 2020).

The mitochondrial genome was assembled using MitoHiFi ( Uliano-Silva *et al.*, 2023).

### Assembly curation

The assembly was decontaminated using the Assembly Screen for Cobionts and Contaminants ( ASCC) pipeline. TreeVal was used to generate the flat files and maps for use in curation. Manual curation was conducted primarily in PretextView and HiGlass ( Kerpedjiev *et al.*, 2018). Scaffolds were visually inspected and corrected as described by Howe *et al*. (2021). Manual corrections included 72 breaks, 83 joins, and removal of 55 haplotypic duplications. This reduced the scaffold count by 54.7%, increased the scaffold N50 by 13.1%, and reduced the total assembly length by 2.6%. The curation process is described at https://gitlab.com/wtsi-grit/rapid-curation. PretextSnapshot was used to generate a Hi-C contact map of the final assembly.

### Assembly quality assessment

The Merqury.FK tool ( Rhie *et al.*, 2020) was run in a Singularity container ( Kurtzer *et al.*, 2017) to evaluate *k*-mer completeness and assembly quality for the primary and alternate haplotypes using the *k*-mer databases ( *k* = 31) computed prior to genome assembly. The analysis outputs included assembly QV scores and completeness statistics.

The genome was analysed using the BlobToolKit pipeline, a Nextflow implementation of the earlier Snakemake version ( Challis *et al.*, 2020). The pipeline aligns PacBio reads using minimap2 ( Li, 2018) and SAMtools ( Danecek *et al.*, 2021) to generate coverage tracks. It runs BUSCO ( Manni *et al.*, 2021) using lineages identified from the NCBI Taxonomy ( Schoch *et al.*, 2020). For the three domain-level lineages, BUSCO genes are aligned to the UniProt Reference Proteomes database ( Bateman *et al.*, 2023) using DIAMOND blastp ( Buchfink *et al.*, 2021). The genome is divided into chunks based on the density of BUSCO genes from the closest taxonomic lineage, and each chunk is aligned to the UniProt Reference Proteomes database with DIAMOND blastx. Sequences without hits are chunked using seqtk and aligned to the NT database with blastn ( Altschul *et al.*, 1990). The BlobToolKit suite consolidates all outputs into a blobdir for visualisation. The BlobToolKit pipeline was developed using nf-core tooling ( Ewels *et al.*, 2020) and MultiQC ( Ewels *et al.*, 2016), with containerisation through Docker ( Merkel, 2014) and Singularity ( Kurtzer *et al.*, 2017).

## Genome sequence report

### Sequence data

**Figure 2.**
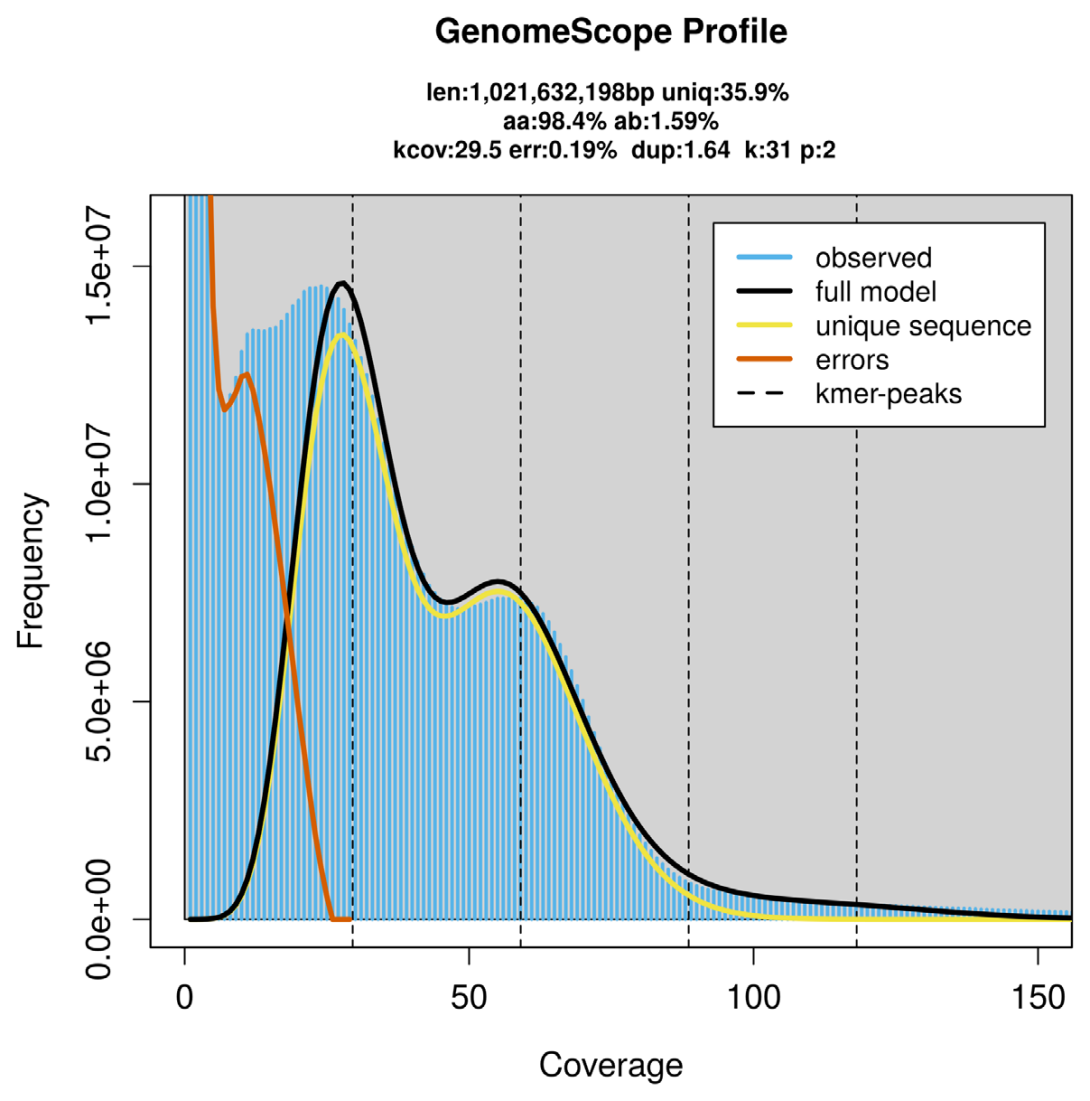
Frequency distribution of *k*-mers generated using GenomeScope2. The plot shows observed and modelled *k*-mer spectra, providing estimates of genome size, heterozygosity, and repeat content based on unassembled sequencing reads.

PacBio sequencing of the *Lamellibrachia columna* specimen generated 66.46 Gb (gigabases) from 7.58 million reads, which were used to assemble the genome. GenomeScope2.0 analysis estimated the haploid genome size at 1 026.13 Mb, with a heterozygosity of 1.59% and repeat content of 64.14% ( Figure 2). These estimates guided expectations for the assembly. Based on the estimated genome size, the sequencing data provided approximately 38× coverage. Hi-C sequencing produced 231.43 Gb from 1 532.68 million reads, which were used to scaffold the assembly. RNA sequencing data were also generated and are available in public sequence repositories. Table 1 summarises the specimen and sequencing details.

**Table 1.** Specimen and sequencing data for BioProject PRJEB68010.

Platform	PacBio HiFi	Hi-C	RNA-seq
**ToLID**	wsLamColu1	wsLamColu1	wsLamColu1
**Specimen ID**	QMOUL0000077	QMOUL0000077	QMOUL0000077
**BioSample (source individual)**	SAMEA12097778	SAMEA12097778	SAMEA12097778
**BioSample (tissue)**	SAMEA12097838	SAMEA12097839	SAMEA12097839
**Instrument**	Sequel IIe	Illumina NovaSeq 6000	Illumina NovaSeq 6000
**Run accessions**	ERR12205280; ERR12205278; ERR12205279; ERR14749934	ERR12245605; ERR12245606	ERR12245607
**Read count total**	7.58 million	1 532.68 million	68.02 million
**Base count total**	66.46 Gb	231.43 Gb	10.27 Gb

### Assembly statistics

The primary haplotype was assembled, and contigs corresponding to an alternate haplotype were also deposited in INSDC databases. The final assembly has a total length of 879.73 Mb in 42 scaffolds, with 706 gaps, and a scaffold N50 of 59.64 Mb ( Table 2).

**Table 2.** Genome assembly statistics.

**Assembly name**	wsLamColu1.1
**Assembly accession**	GCA_963662155.1
**Alternate haplotype accession**	GCA_963667965.1
**Assembly level**	chromosome
**Span (Mb)**	879.73
**Number of chromosomes**	15
**Number of contigs**	748
**Contig N50**	1.93 Mb
**Number of scaffolds**	42
**Scaffold N50**	59.64 Mb
**Organelles**	Mitochondrion: 16.78 kb

**Figure 3.**
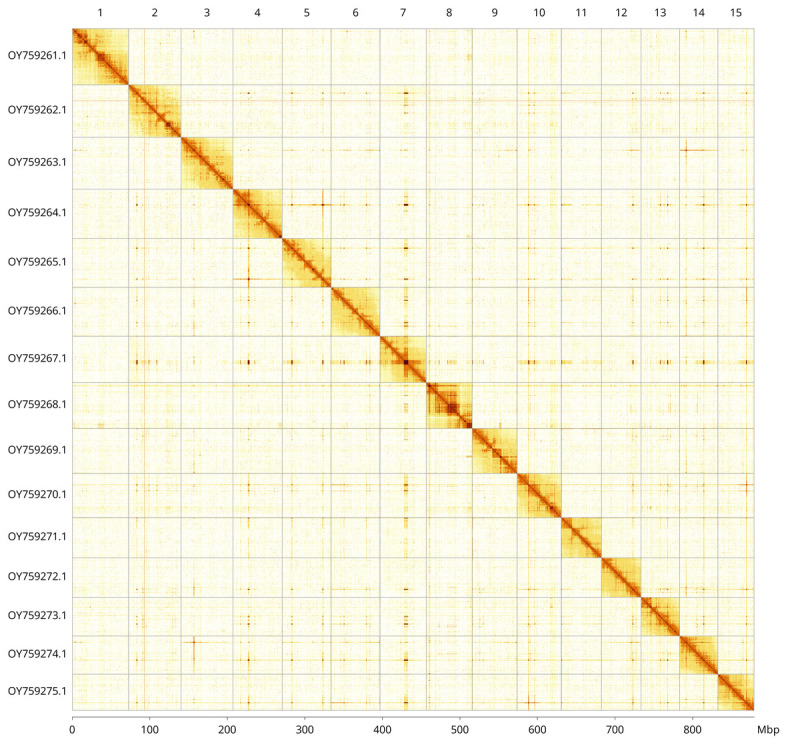
Hi-C contact map of the *Lamellibrachia columna* genome assembly. Assembled chromosomes are shown in order of size and labelled along the axes, with a megabase scale shown below. The plot was generated using PretextSnapshot. An interactive version of the contact map may be viewed in HiGlass.

Most of the assembly sequence (99.96%) was assigned to 15 chromosomal-level scaffolds. These chromosome-level scaffolds, confirmed by Hi-C data, are named according to size ( Figure 3; Table 3).

**Table 3.** Chromosomal pseudomolecules in the primary genome assembly of *Lamellibrachia columna* wsLamColu1.

INSDC accession	Molecule	Length (Mb)	GC%
OY759261.1	1	72.70	40.50
OY759262.1	2	67.76	40.50
OY759263.1	3	66.82	40.50
OY759264.1	4	63.57	40.50
OY759265.1	5	63.17	40.50
OY759266.1	6	63.05	40.50
OY759267.1	7	59.64	40.50
OY759268.1	8	59.20	40.50
OY759269.1	9	58.07	40.50
OY759270.1	10	56.89	40.50
OY759271.1	11	51.82	40.50
OY759272.1	12	50.84	40.50
OY759273.1	13	49.93	40.50
OY759274.1	14	49.26	40.50
OY759275.1	15	46.62	40.50

The mitochondrial genome was also assembled (length 16.78 kb, OY759276.1). This sequence is included as a contig in the multifasta file of the genome submission and as a standalone record.

### Assembly quality metrics

**Figure 4.**
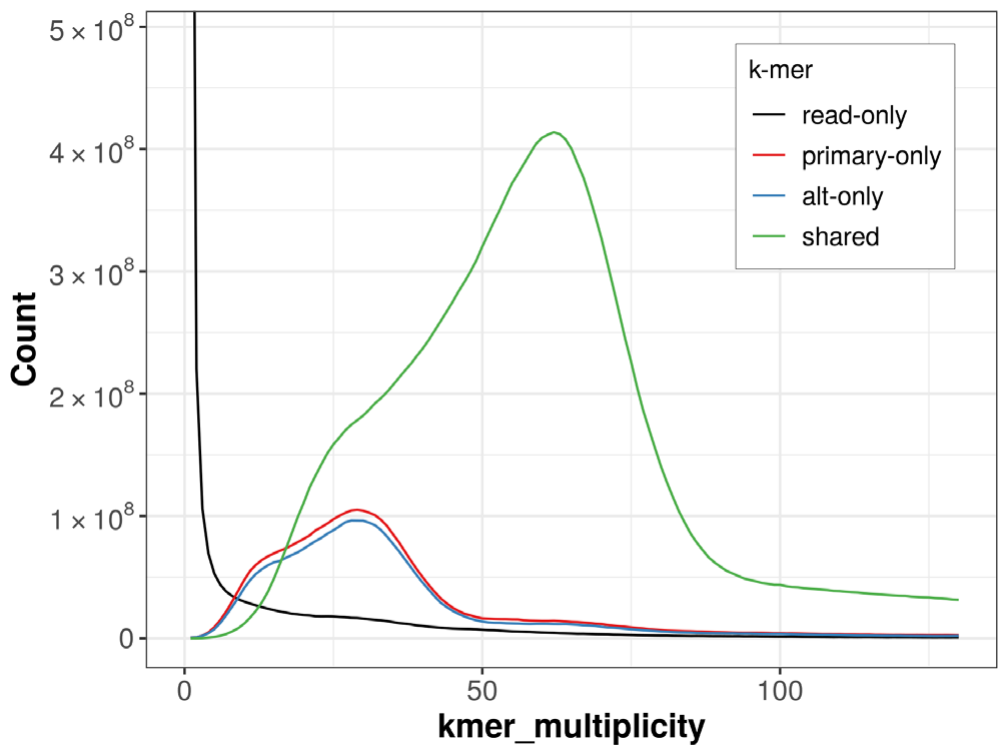
Evaluation of *k*-mer completeness using MerquryFK. This plot illustrates the recovery of *k*-mers from the original read data in the final assemblies. The horizontal axis represents *k*-mer multiplicity, and the vertical axis shows the number of *k*-mers. The black curve represents *k*-mers that appear in the reads but are not assembled. The green curve corresponds to *k*-mers shared by both haplotypes, and the red and blue curves show *k*-mers found only in one of the haplotypes.

The combined primary and alternate assemblies achieve an estimated QV of 54.0. The *k*-mer completeness is 76.92% for the primary assembly, 75.61% for the alternate haplotype, and 94.12% for the combined assemblies ( Figure 4).

**Figure 5.**
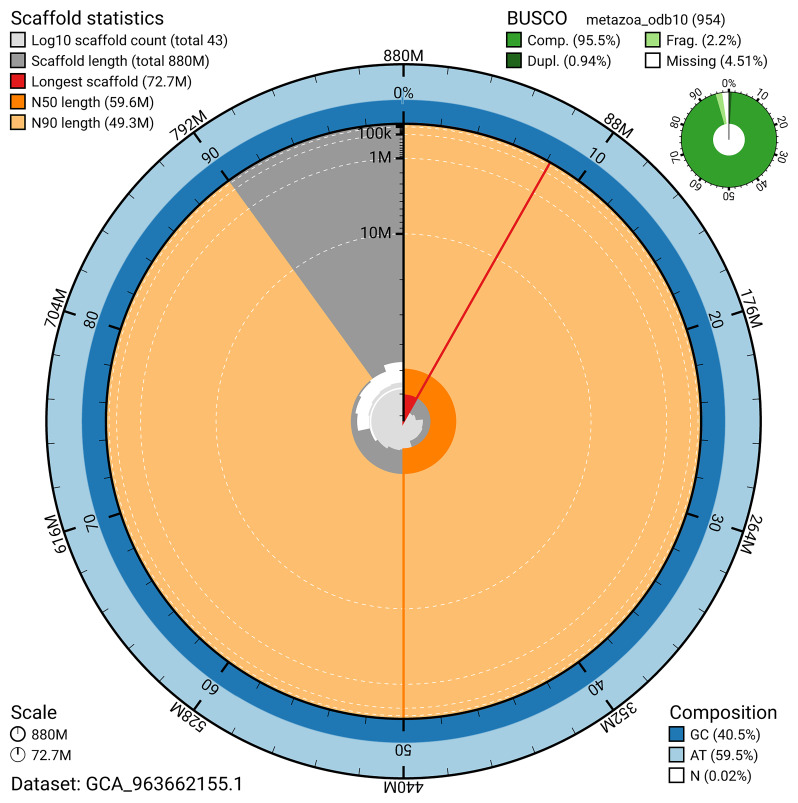
Assembly metrics for wsLamColu1.1. The BlobToolKit snail plot provides an overview of assembly metrics and BUSCO gene completeness. The circumference represents the length of the whole genome sequence, and the main plot is divided into 1 000 bins around the circumference. The outermost blue tracks display the distribution of GC, AT, and N percentages across the bins. Scaffolds are arranged clockwise from longest to shortest and are depicted in dark grey. The longest scaffold is indicated by the red arc, and the deeper orange and pale orange arcs represent the N50 and N90 lengths. A light grey spiral at the centre shows the cumulative scaffold count on a logarithmic scale. A summary of complete, fragmented, duplicated, and missing BUSCO genes in the set is presented at the top right. An interactive version of this figure can be accessed on the BlobToolKit viewer.

**Figure 6.**
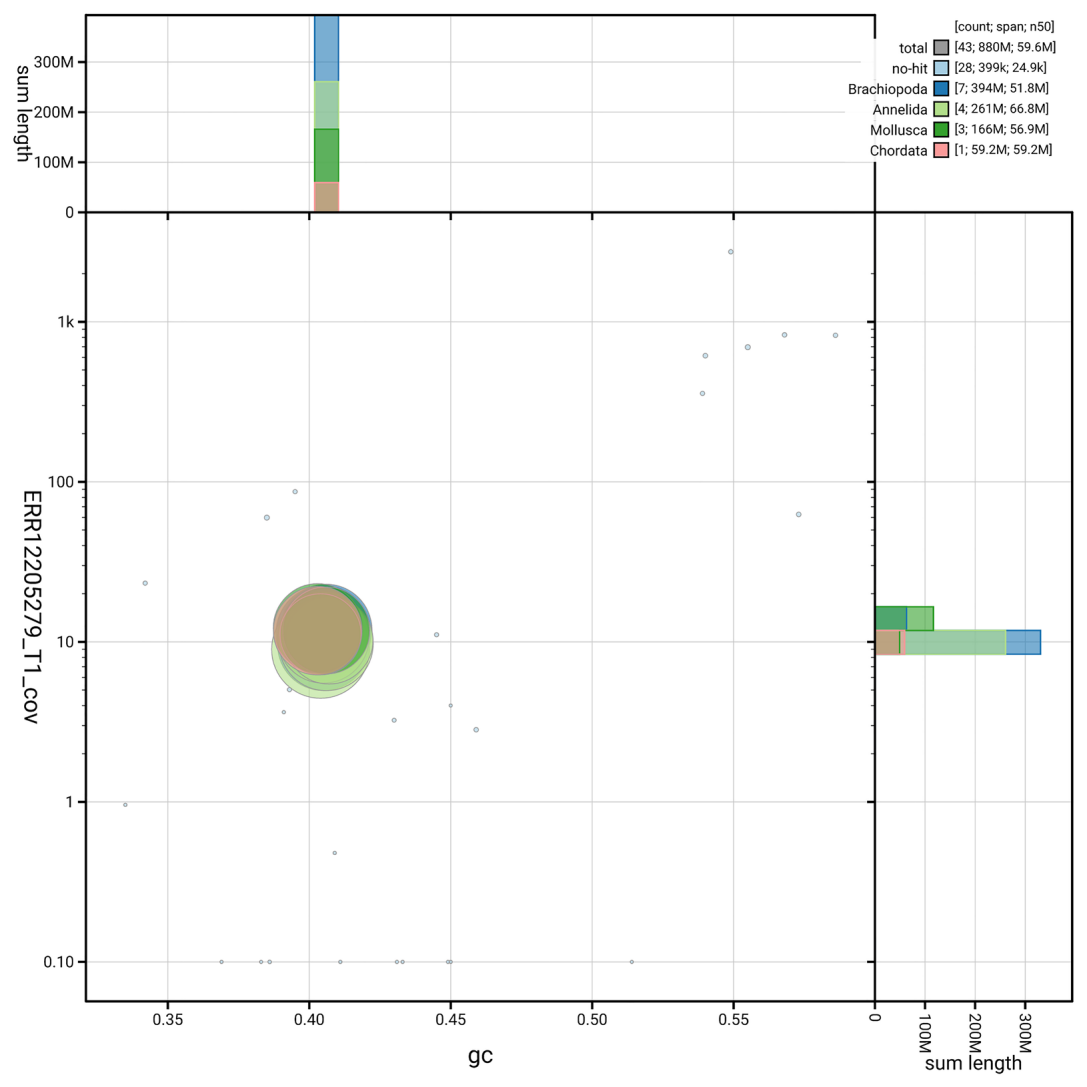
BlobToolKit GC-coverage plot for wsLamColu1.1. Blob plot showing sequence coverage (vertical axis) and GC content (horizontal axis). The circles represent scaffolds, with the size proportional to scaffold length and the colour representing phylum membership. The histograms along the axes display the total length of sequences distributed across different levels of coverage and GC content. An interactive version of this figure is available on the BlobToolKit viewer.

BUSCO v.5.5.0 analysis using the reference set ( *n* = ) identified % of the expected gene set (single = %, duplicated = %). The snail plot in Figure 5 summarises the scaffold length distribution and other assembly statistics for the primary assembly. The blob plot in Figure 6 shows the distribution of scaffolds by GC proportion and coverage.

Table 4 lists the assembly metric benchmarks adapted from Rhie *et al.* (2021) and the Earth BioGenome Project Report on Assembly Standards September 2024. The EBP metric, calculated for the primary assembly, is **6.C.Q53**, meeting the recommended reference standard.

**Table 4.** Earth Biogenome Project summary metrics for the *Lamellibrachia columna* assembly.

Measure	Value	Benchmark
EBP summary (primary)	6.C.Q53	6.C.Q40
Contig N50 length	1.93 Mb	≥ 1 Mb
Scaffold N50 length	59.64 Mb	= chromosome N50
Consensus quality (QV)	Primary: 53.7; alternate: 54.4; combined: 54.0	≥ 40
*k*-mer completeness	Primary: 76.92%; alternate: 75.61%; combined: 94.12%	≥ 95%
BUSCO	C:94.6%[S:93.8%,D:0.8%];F:3.0%,M:2.4%,n:954	S > 90%; D < 5%
Percentage of assembly assigned to chromosomes	99.96%	≥ 90%

**Notes:** EBP summary uses log10(Contig N50); chromosome-level (C) or log10(Scaffold N50); Q (Merqury QV). BUSCO: C=complete; S=single-copy; D=duplicated; F=fragmented; M=missing; n=orthologues

## Genome annotation report

The *Lamellibrachia columna* genome assembly (GCA_963662155.1) was annotated by Ensembl at the European Bioinformatics Institute (EBI). This annotation includes 36 475 transcribed mRNAs from 21 983 protein-coding and 4 157 non-coding genes. The average transcript length is 14 550.97 bp, with an average of 1.40 coding transcripts per gene and 7.12 exons per transcript. For further information about the annotation, please refer to the Ensembl annotation page.

## Wellcome Sanger Institute – Legal and Governance

The materials that have contributed to this genome note have been supplied by a Tree of Life collaborator. The Wellcome Sanger Institute employs a process whereby due diligence is carried out proportionate to the nature of the materials themselves, and the circumstances under which they have been/are to be collected and provided for use. The purpose of this is to address and mitigate any potential legal and/or ethical implications of receipt and use of the materials as part of the research project, and to ensure that in doing so we align with best practice wherever possible.

The overarching areas of consideration are:

- Ethical review of provenance and sourcing of the material
- Legality of collection, transfer and use (national and international)

Each transfer of samples is undertaken according to a Research Collaboration Agreement or Material Transfer Agreement entered into by the Tree of Life collaborator, Genome Research Limited (operating as the Wellcome Sanger Institute) and in some circumstances other Tree of Life collaborators.

## Data Availability

European Nucleotide Archive: Lamellibrachia columna. Accession number PRJEB68010. The genome sequence is released openly for reuse. The *Lamellibrachia columna* genome sequencing initiative is part of the Aquatic Symbiosis Genomics Project (PRJEB43743) and Sanger Institute Tree of Life Programme (PRJEB43745). All raw sequence data and the assembly have been deposited in INSDC databases. Raw data and assembly accession identifiers are reported in Table 1 and Table 2. Production code used in genome assembly at the WSI Tree of Life is available at https://github.com/sanger-tol. Table 5 lists software versions used in this study.

## Author information

Contributors are listed at the following links:

- Members of the Wellcome Sanger Institute Tree of Life Management, Samples and Laboratory team
- Members of Wellcome Sanger Institute Scientific Operations – Sequencing Operations
- Members of the Wellcome Sanger Institute Tree of Life Core Informatics team
- Members of the EBI Aquatic Symbiosis Genomics Data Portal Team
- The Aquatic Symbiosis Genomics Project leadership
