# Supplementary material for: The chromosomal genome sequence of the tubeworm, Lamellibrachia columna Southward, 1991 (Sabellida: Siboglinidae)
Source: Wellcome Open Res. 2026 Feb 16;11:127. [Version 1] doi: 10.12688/wellcomeopenres.25860.1 (PMC13080331; doi:10.12688/wellcomeopenres.25860.1)
Supplement: Peer review history

# Reviewer report: Reviewer response for version 1

**DOI:** 10.21956/wellcomeopenres.28463.r152196

**Published:** 2026-04-14

**Authors:** Thomas D. Lewin

**Affiliations:** 1 Queen Mary University of London School of Biological and Behavioural Sciences (Ringgold ID: 646267), London, England, UK

Sun et al. present a reference assembly for the tubeworm *Lamellibrachia columna*. As a deep-sea annelid with chemosynthetic endosymbiotic bacteria, this is a key species for those studying symbiosis, deep-sea environments, and annelid evolution.

 The note is of the standard format produced by the Tree of Life Programme. Methods and protocols are very well documented and the genome is extremely high quality. It also has a more extensive introduction to the species than many other genome notes which gives a nice pointer to why this particular species is interesting and how the genome could be of use. I have no major concerns about the manuscript and only a couple of minor queries.

 First, regarding the BlobToolKit output in Figure 6: if I am reading it correctly, only four of the 43 scaffolds have a phylum membership of Annelida while 7 are Brachiopoda, 3 Mollusca, and 28 have no hit. I expect this kind of result with very understudied phyla that may be unrepresented in databases but I am quite surprised to see it for an annelid. Do the authors have an explanation for this result?

 Second, could the authors expand on very specific terms like vestimentum and opisthosoma to make the background more accessible to those who are interested in the genome but not in the annelid field.  

 Finally, I agree with the remarks of the first reviewer who noted:

1. The absence of BUSCO data in the text and a discrepancy in the BUSCO results reported in Table 4 and Figure 5
2. The absence of an explanation of the genome annotation procedure. I have raised this issue regarding previous genome notes and think it is an important point to be corrected.

Overall, this is a high-quality genome that will be of significant benefit to the field.

Are sufficient details of methods and materials provided to allow replication by others?

Yes

Is the rationale for creating the dataset(s) clearly described?

Yes

Are the datasets clearly presented in a useable and accessible format?

Yes

Are the protocols appropriate and is the work technically sound?

Yes

Reviewer Expertise:

Evolutionary genomics of spiralians

I confirm that I have read this submission and believe that I have an appropriate level of expertise to confirm that it is of an acceptable scientific standard.

# Reviewer report: Reviewer response for version 1

**DOI:** 10.21956/wellcomeopenres.28463.r152199

**Published:** 2026-04-06

**Authors:** Erich M. Schwarz

**Affiliations:** 1 Cornell University, Ithaca, New York, USA

Sun et al. provide a chromosomally scaffolded genome sequence for Lamellibrachia columna.  Their report is generally satisfactory.  However, I do have a few corrections to suggest.

 In the Grant Information section: "(grant number 2023HWYQ-101), the Taishan Scholars Program" might be better written as "(grant number 2023HWYQ-101) and the Taishan Scholars Program".

 In the legend of Figure 1, "Samagi Bay" appears to be a typographical error; it should probably read "Sagami Bay".

 In the 'Assembly quality metrics' section of the Results, the BUSCO results need to be properly filled in.  Presently, they read: "BUSCO v.5.5.0 analysis using the [which?] reference set (n = ) identified % of the expected gene set (single = %, duplicated = %)."  If I were to try to correct this from the numbers given in Figure 5, then I would rewrite this as: "BUSCO v.5.5.0 analysis using the metazoa_odb10 reference set (n = 954) identified 96.4% of the expected gene set (single = 95.5%, duplicated = 0.94%)."  However, these numbers have another problem; they are inconsistent with the BUSCO values given in Table 4!  The authors should review the BUSCO values given in Figure 5 versus Table 4, explain any apparent discrepancies, correct any real discrepancies, and fill in the sentence in 'Assembly quality metrics' appropriately.

 In the Genome Annotation Report, the authors write: "The Lamellibrachia columna genome assembly (GCA_963662155.1) was annotated by Ensembl at the European Bioinformatics Institute (EBI)."  They later refer readers to the Ensembl web page https://beta.ensembl.org/species/0d55dde9-c871-4112-88c2-3e8491b8d41d for details.  However, this web page provides no details about *how* these annotations were generated.  There must be, somewhere, some reference or web manual by Ensembl that explains in detail how these gene predictions were made by Ensembl.  Could the authors please provide a citation to these detailed prediction methods?

Are sufficient details of methods and materials provided to allow replication by others?

Partly

Is the rationale for creating the dataset(s) clearly described?

Yes

Are the datasets clearly presented in a useable and accessible format?

Yes

Are the protocols appropriate and is the work technically sound?

Yes

Reviewer Expertise:

Nematode genome/transcriptome analysis

I confirm that I have read this submission and believe that I have an appropriate level of expertise to confirm that it is of an acceptable scientific standard.
